# Development of a Polyvinylidene fluoride–based membrane incorporating magnetic iron–nickel alloy for vacuum membrane distillation desalination

**DOI:** 10.1038/s41598-026-52863-3

**Published:** 2026-05-19

**Authors:** Eman Farag, Norhan Nady, Elham El-Zanati

**Affiliations:** 1 Chemical Engineering and Pilot Plant Department, Engineering Research Division, National Research Centre, 33 Bohouth St. (Former El Tahrir St.), Dokki, P.O. box 12622, Giza, Egypt; 2 Polymeric Materials Research Department, Advanced Technology and New Materials Research Institute (ATNMRI), City of Scientific Research and Technological Applications (SRTA-City), Borg El-Arab City, 21934 Alexandria Egypt

**Keywords:** Starfish-like filler, PVDF membrane, Vacuum membrane distillation, Water desalination, Engineering, Materials science

## Abstract

Novel mixed a starfish-like shaped magnetic iron-nickel alloy with Polyvinylidene fluoride (PVDF) matrix membranes were developed for water desalination using vacuum membrane distillation (VMD). This study highlights the alloy’s unique morphology that coated by the hydrophobic polymer, which enhances water vapor transport through innovative pore formation and its permanent magnetic properties, distinguishing it from existing research. The membranes were characterized using scanning electron microscopy (SEM), Energy Dispersive X‑ray (EDX) analysis and mapping, Fourier Transform Infrared Spectroscopy with Attenuated Total Reflection (FTIR-ATR), and Thermal Gravimetric Analysis (TGA), along with measurements of Liquid Entry Pressure (LEP), static water contact angle, tensile strength, thickness, roughness, porosity, pore size and pore size distribution. Performance tests in a VMD system showed that the iron–nickel alloy increased membrane productivity by 47% compared to pristine PVDF membranes. The 0.2 wt% alloy with 14% PVDF achieved the highest porosity (74.32%) and flux (29.1 kg/m²·h), balancing surface roughness and structural integrity. In contrast, higher polymer content (18 wt% PVDF) negatively impacted porosity and led to performance trade-offs. Thus, this study emphasizes the critical interplay between porosity, roughness, thickness, and the magnetic properties of the alloy in optimizing membrane performance for VMD applications.

## Introduction

The rapidly increasing global demand for freshwater, driven by climate change and population growth, highlights the urgent need for the development of innovative desalination technologies. Among the emerging approaches, membrane distillation (MD) has gained considerable attention as a compelling method due to its energy efficiency and capability to function at low temperatures^1,2^.

This study is motivated by the necessity to develop advanced membrane materials that not only enhance separation efficiency but also address these pressing issues. By integrating low-cost starfish-like shaped magnetic iron-nickel alloy into Polyvinylidene fluoride (PVDF) membranes, we aim to improve water vapor transport through innovative pore formation. Additionally, the alloy’s permanent magnetic properties may provide new avenues for mitigating fouling (as will be studied in future work) and enhancing membrane longevity, thereby contributing to more sustainable water desalination solutions.

In this framework, integrating diverse filler materials into polymer membranes emerges as a pioneering strategy to enhance performance. Mixed matrix membranes (MMMs), which amalgamate a polymer matrix with inorganic or organic fillers, exploit the beneficial properties of both constituents to elevate separation efficacy. Recent investigations have examined a variety of fillers, such as zeolites^3^, graphene oxide (GO)^4^, metal-organic frameworks (MOFs)^5^, carbon nanotubes (CNTs)^5^, metal oxides such as titanium dioxide (TiO_2_)^4,5^, graphene nanotubes^6^, nanoplates^7^, and recently metal alloys^8^. These fillers can enhance membrane properties such as hydrophobicity, mechanical strength, thermal stability, and antifouling characteristics. The use of iron-nickel magnetic alloys as a filler in PVDF matrices offers dual benefits: magnetic response behavior that can facilitate membrane regeneration and improved mechanical properties. This innovative approach has the potential to optimize the membrane’s hydrophobicity, thus enhancing flux rates and salt rejection efficiency.

In MD, a microporous hydrophobic membrane acts as a barrier to prevent interaction between the aqueous feed stream and the resultant water vapor^9^. The evolution of MD technology within separation processes has garnered substantial attention, owing to its advantages such as minimal operating pressure, elevated water flux, and high rejection rates for non-volatile compounds, inorganic ions, and macromolecules. Notably, vacuum membrane distillation (VMD) offers enhanced permeation flux, improved thermal efficiency, and compatibility with renewable energy sources like geothermal power, solar energy, and waste heat streams, rendering it superior to earlier MD formats^10,11^.

The performance of MD fundamentally relies on the characteristics of the membrane, which must facilitate selective water vapor transport while withstanding harsh saline conditions. PVDF has emerged as an exceptionally effective material for membrane fabrication due to its remarkable operability and customizable formation capabilities^12,13^. Noted for its thermal stability and chemical resilience, PVDF is a hydrophobic polymer that encounters significant limitations in permeate flux during continuous operation^14,15^. To enhance the hydrophobicity and overall performance of PVDF membranes, extensive research has focused on methods such as blending with (super)hydrophobic polymers, surface grafting, coating, and incorporating inorganic fillers^16–19^. Additionally, the strategic design of PVDF membranes for MD should prioritize superior durability, consistent stability during use, and commendable mechanical strength. Numerous studies have highlighted the benefits of incorporating inorganic nanoparticles/nanofillers into polymer matrices to enhance mechanical, thermal, and chemical stability, with an emphasis on increasing porosity, surface roughness, and water vapor flux^20–24^.

Iron-nickel alloys represent a novel category of advanced materials with exceptional attributes, such as superior magnetic, electrical, and selective adsorption properties, relevant for water treatment applications^25,26^. Their inclusion can influence phase inversion kinetics and membrane precipitation rates during solvent exchange, facilitating the development of macro-voids in the membrane matrix’s sub-layer, subsequently improving water flux. Moreover, owing to their superior adsorptive characteristics, these alloys can significantly enhance the selective adsorption of ions, thereby improving rejection capabilities^8,27^.

This study introduces innovative mixed matrix membranes developed from a PVDF matrix integrated with a magnetic iron–nickel alloy, specifically evaluated for water desalination using VMD. The membranes were systematically characterized through various analytical techniques, including scanning electron microscopy (SEM), Energy Dispersive X‑ray (EDX) analysis and mapping, Fourier transform infrared spectroscopy with attenuated total reflection (FTIR-ATR), and thermogravimetric analysis (TGA). Key membrane properties such as Liquid Entry Pressure (LEP), static water contact angle, tensile strength, thickness, surface roughness, porosity, pore size and pore size distribution were also analyzed. The desalination performance of the fabricated membranes was tested in a VMD system established at the National Research Centre (NRC) in Egypt.

While the Fe–Ni alloy/filler used was characterized by its hydrophilicity, it was embedded within a hydrophobic polymer, limiting direct contact with the membrane vapor. The primary function of the filler is to facilitate pore formation and enhance water vapor attraction into the membrane pores due to its magnetic properties. These unique characteristics distinguish our work from existing literature, highlighting the potential of this material combination to advance membrane distillation technologies. Overall, this paper elucidates the fabrication and characterization of the mixed PVDF matrix membrane infused with a magnetic iron-nickel alloy, assessing its efficacy in desalination via VMD. The findings are expected to significantly advance membrane technologies in the pursuit of sustainable freshwater solutions.

## Materials and methods

### Materials

Polyvinylidene fluoride (PVDF, 99% assay) powder, 1-Methyl − 2- pyrrolidinone (NMP, 99% assay), and absolute ethanol (assay ≥ 99%) were acquired from Fisher, UK. Lithium chloride (LiCl, 99% purity) used as a pore-forming was obtained from Sigma Aldrich, USA. Ethylene glycol (EG, assay ≥ 99%, boiling temperature 197.3 °C) was purchased from Fisher Scientific, Germany. The chemicals employed for alloy synthesis included Nickel Chloride Hexahydrate (NiCl₂·6 H₂O, 98%) and Ferrous Chloride Tetrahydrate (FeCl₂·4 H₂O, 99.99%) as metal ion sources, procured from Sigma, Germany. Hydrazine Hydrate (N₂H₄·H₂O, 99%) served as the reducing agent and was sourced from Fisher, UK. Additionally, Sodium Hydroxide (NaOH, 98%) was acquired from Trading Dynamic Co. (TDC) in Egypt.

### Methods

#### Synthesis of iron-nickel alloys

Nanocrystalline filler samples of iron-nickel alloys were previously synthesized as presented in reference^26^. Aqueous solutions of iron and nickel salts were prepared with molar ratios of 1:9 using distilled water as the solvent. The resulting solution was vigorously stirred using a temperature-controlled magnetic stirrer at 1400–1600 rpm while maintaining the temperature between 95 and 98 °C. A second solution consisting of warm aqueous hydrazine (N₂H₄·H₂O, 99 wt%) and aqueous sodium hydroxide (0.1 M) with a pH of 12.8 was then mixed with the first solution. The final black particles were subsequently isolated using a magnetic separation, thoroughly washed with distilled water until approaching a neutral pH 7, and dried in a vacuum oven at 35 °C for 24 h.

#### Preparation of nanocomposite membrane for MD application

The nanocomposite membranes were prepared by the phase inversion method. The dope solution was prepared by dissolving Polyvinylidene fluoride (PVDF) polymer in 1-Methyl − 2- pyrrolidinone (NMP) with the addition of Ethylene glycol (EG) and Lithium chloride (LiCl; 4 mg/100 g dope solution) as pore formers in the dope solution. This dope solution was stirring mechanically in a glassy beaker using overhead stirrer (model OSD-10, LK LABKOREA) at 400 rpm for 4 h at 50 °C. For better dispersion of iron-nickel alloy and breaking up its aggregates, it was dispersed in 40 wt% of the used total solvent amount and the solutions were sonicated for 30 min using an intelligent ultrasonic processor (model: JY92- IIDN, HUXI, China). Then the dispersed iron-nickel alloy was mixed in the prepared PVDF dope. The obtained homogeneous dops were cast onto clean and dry glass plates using a casting knife with a thickness of 400 μm. Then, the spread sheets were immediately immersed in distillate water as non-solvent coagulation bath at room temperature (25 °C) for two hours. The formed membranes were kept in new containers with fresh distillate water for one day to remove any soluble components in the membrane structures. The membranes were dried at room temperature (25 °C). The composition of the used casting solutions is given in Table 1.

**Table 1:** The membrane code and its dope composition (wt.%) of the fabricated membranes.

Membrane Code	Membrane Composition (wt.% of total dope weight)				
	**PVDF**	**NMP**	**EG**	**LiCl**	**Iron-nickel alloy**
F0	14	85	1	0.004	Pristine PVDF
F1	14	85	1	0.004	0.01
F2	14	85	1	0.004	0.1
F3	14	85	1	0.004	0.2
F4	18	81	1	0.004	0.01

#### Membrane thickness

The average thickness was determined by taking ten measurements at various locations on three distinct membrane samples, using a micrometer (range: 0–25 mm, precision: 2 μm, HDT, China). The standard deviation was calculated and added as error bar.

#### Membrane surface roughness

A surface roughness analyzer (SJ-201P, Japan) was used to evaluate the membrane surface roughness. Prior to measurement, the instrument was calibrated using a glass plate employed to fix the membrane samples. For each condition, seven measurements were recorded from two different membrane samples, and the average roughness value was reported.

#### Fourier Transform Infrared equipped by Attenuated Total Reflection (FTIR-ATR) analysis

Fourier Transform Infrared (FTIR) spectra equipped with Attenuated Total Reflection (ATR) were recorded using an ALPHA II FTIR spectrometer (Bruker Optik GmbH, Germany). The spectra were collected over the range of 4000–400 cm⁻¹ with a spectral resolution of 4 cm⁻¹.

#### Thermal gravimetric Analysis (TGA)

The thermal stability of the F0 and F3 membranes was evaluated by thermogravimetric analysis (TGA), with mass loss reported as a percentage of the initial sample weight. Measurements were conducted using a Themys One+ thermogravimetric analyzer over a temperature range of 30–900 °C at a heating rate of 10 °C min⁻¹, under a nitrogen atmosphere with a flow rate of 50 mL min⁻¹.

#### Membrane porosity

The porosity of the membranes was determined using the gravimetric method, in which porosity is defined as the ratio of the pore volume to the total membrane volume. Ethanol was employed as the wetting liquid to ensure complete penetration into the membrane pores^28^. After removing excess ethanol from the surface and allowing residual ethanol to evaporate, the weight of the wetted membrane (m_w_, g) was recorded. The membranes were then completely air-dried under ambient conditions and reweighed to obtain the dry mass (m_d_, g). For both wet and dry states, the average weight of three membrane samples was used. Membrane porosity was calculated according to Eq. (1)^28,29^.

1 $$\:\epsilon\:\:\left(\%\right)=\:\frac{\frac{{m}_{w}-{m}_{d}}{{\rho\:}_{e}}}{\left[\frac{{m}_{w}-{m}_{d}}{{\rho\:}_{e}}\right]+\:\frac{{m}_{d}}{{\rho\:}_{p}}}\:\:\mathrm{x}\:100$$

Where ε is the membrane porosity (%), m_*w*_ is the weight of wet membrane (g), m_*d*_ is the weight of dry membrane (g), $\:{\rho}_{p}$ is the density of the polymer (g/cm^3^), and $\:{\rho}_{e}$ is the density of water (g/cm^3^).

The pore size distribution of F3 was determined from scanning electron microscopy (SEM) micrographs using the ImageJ software, calibrated according to the scale bars. In total, over 1,000 pores were measured to ensure the reliability of the statistical data. To enhance the accuracy of the analysis, outliers were identified and removed using the interquartile range method prior to conducting further statistical evaluations.

#### Scanning Electron Microscopy (SEM) imaging and Energy Dispersive X‑ray (EDX) analysis

Scanning Electron Microscopy (SEM) was used to examine the morphological features and surface topography of the fabricated membranes. Prior to imaging, the dried samples were sputter-coated with a thin gold layer to improve electrical conductivity. High-resolution micrographs were obtained using a JEOL 5410 scanning electron microscope operated at an accelerating voltage of 10 kV. The chemical compositions were determined by an area analysis using energy-dispersive X-ray spectroscopy (EDX) system equipped with SEM.

#### Static water contact angle (membrane wettability)

To evaluate the surface wettability and hydrophilicity of the PVDF/iron–nickel alloy membranes, water contact angle measurements were performed. Small droplets (7 µL) of deionized water were carefully deposited onto the membrane surfaces, and the resulting droplet images were captured using a digital camera. The contact angles of the pristine PVDF and PVDF/iron–nickel composite membranes were determined using the sessile drop method with SCA20 and OCA15EC instruments at the National Research Centre (MNRC).

#### Membrane mechanical strength

The mechanical properties of the flat membranes were evaluated using a computer-controlled universal testing machine (WDW-5, HST Corp.) at ambient temperature. The membrane sheets were firmly clamped at both ends with an initial gauge length of 50 mm. All tests were conducted at a constant crosshead speed of 50 mm min⁻¹. The reported mechanical properties correspond to the average values obtained from at least three independent membrane samples.

#### Determination of Liquid Entry Pressure (LEP)

Liquid Entry Pressure (LEP), defined as the minimum pressure required for liquid water to permeate the membrane, was measured for every fabricated membrane using a straightforward setup built in our laboratory. The arrangement comprises a nitrogen gas cylinder, the membrane testing chamber, and a pressure indicator. Initially, a dry membrane sample was placed into the test chamber. The membrane testing chamber was filled with distilled water, and nitrogen gas was gradually introduced to the feed side, progressively increasing the water’s hydrostatic pressure and driving it toward the membrane surface. The pressure was incremented in small steps until the first water droplet appeared on the opposite side of the membrane. The corresponding pressure reading on the gauge at that moment was recorded as the LEP. This procedure was conducted in triplicate for each membrane, and the three measurements were averaged to obtain the final LEP value.

#### Vacuum Membrane Distillation (VMD) system

**Fig. 1:**
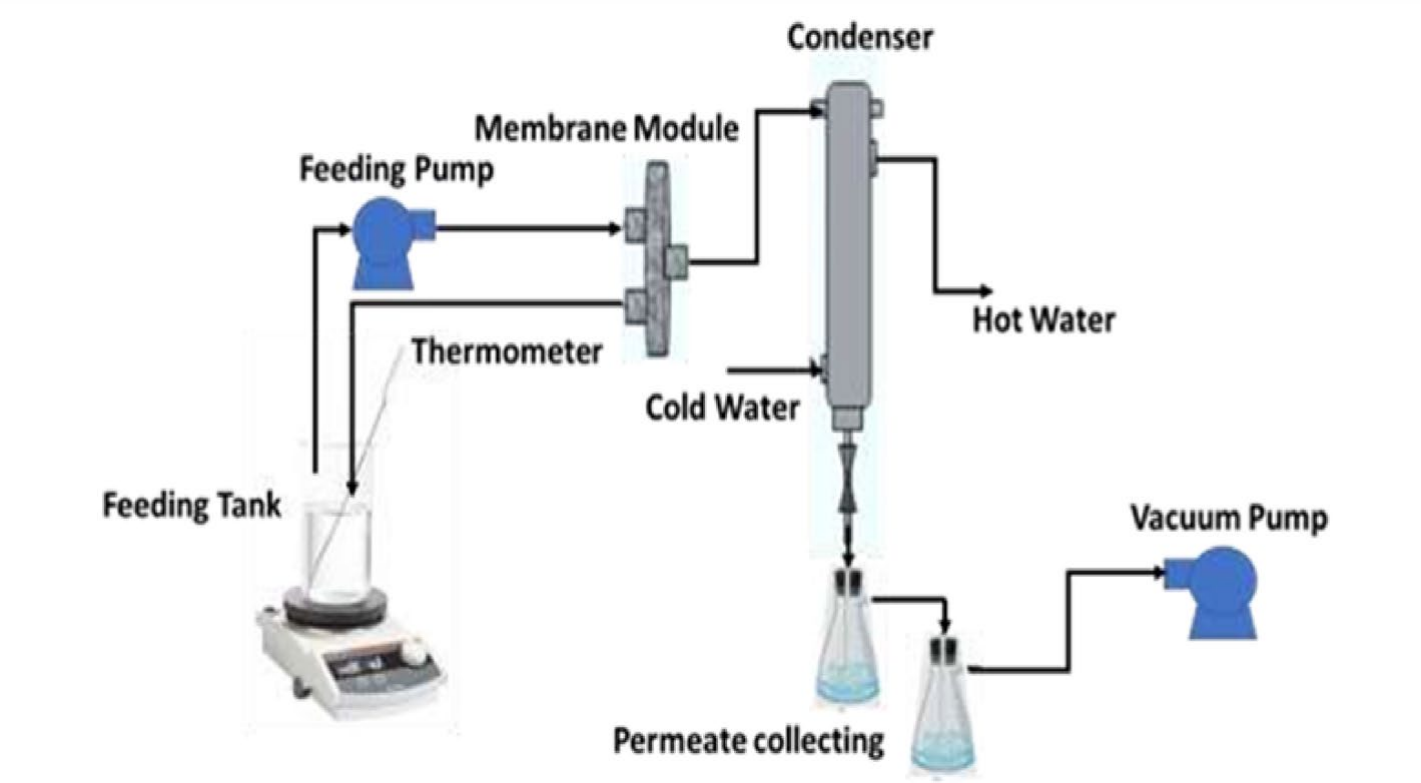
Schematic diagram of the used vacuum membrane distillation (VMD system^29^ established at the National Research Centre (NRC) in Egypt.

The fabricated membranes were systematically evaluated for their suitability as hydrophobic porous membranes using a VMD unit at the National Research Centre, Egypt, as illustrated in Fig. 1. Each membrane was securely mounted in a custom-designed membrane cell with an effective area of 16 cm² and connected to the feed and permeate sides via silicone tubing. An aqueous NaCl solution (3.5 wt%) was prepared and introduced into the feed tank, where it was heated to 70 °C using a continuously stirred hot plate (MSH-20D, DAIHAN Scientific Co., Korea). The heated feed solution was circulated through the membrane cell at a flow rate of 15 cm³ s⁻¹ (the calculated feed pressure is about 0.07 bar) using a peristaltic pump (YT600-1JKZ35, Longer Pump Co., China). A vacuum of 0.5 bar relative to atmospheric pressure applied to the permeate side using a vacuum pump (SH-V40, SH Scientific, Korea). This setup facilitated the extraction of water vapor, which was subsequently condensed in a tube condenser cooled with circulating water maintained at 10 °C. The permeate flux and electrical conductivity were measured at 30-minute experimental time. For each membrane type, four runs were done and the average permeate and rejection were calculated. To maintain a constant feed concentration throughout the experiment, an equivalent volume of distilled water was periodically added to the feed tank. The permeate flux was calculated according to Eq. (2).

2 $$\:{J}_{w}=\raisebox{1ex}{$V$}\!\left/\:\!\raisebox{-1ex}{$A$}\right.$$

Where $\:{J}_{w}\:$is the volumetric membrane flux (L m⁻² h⁻¹), $\:V\:$is the volumetric flow rate of the permeate (L h⁻¹), and $\:A\:$is the effective membrane area (m²). The salt rejection (SR) was calculated using Eq. (3):

3 $${\text{SR }}={\text{ }}\left( {{\mathrm{1}}\, - \,{{\mathrm{C}}_{\mathrm{P}}}/{{\mathrm{C}}_{\mathrm{F}}}} \right) * {\mathrm{1}}00$$

Where C_P_ is the final salt concentration of the permeate stream and C_F_ is the initial salt concentration of permeate.

## Results and discussion

The performance of polyvinylidene fluoride/Iron-Nickel (PVDF/Fe–Ni) nanocomposite membranes in vacuum membrane distillation (VMD) is governed by a complex interplay of structural, morphological, mechanical, thermal, and surface properties. Optimizing these parameters is essential for achieving high flux, effective salt rejection, and mechanical durability.

In cast membrane studies, the majority of polymer concentrations commonly fall in the 14–18 wt% range. Given the properties of PVDF, most researchers aim for the lower end of this spectrum (around 14 wt%) to optimize processability and morphology^7^. To systematically explore how polymer loading influences membrane structure and performance, we deliberately increased the concentration to 18 wt%. This higher concentration enables us to study the effect of polymer content on the resulting mixed-matrix membrane and to assess whether thicker skin, different porosity, or altered demixing behavior emerge (the coagulation kinetics “solvent–non-solvent exchange rate” can shift with PVDF loading, potentially slowing demixing and producing a denser, thicker top layer that reads as increased thickness) at the upper end of the practical concentration window^30^.

Two pore-forming agents, ethylene glycol (EG) and lithium chloride (LiCl) (as detailed in Table 1), within the dope solution have been used. The rationale for incorporating both agents lies in their complementary roles in modulating membrane properties. EG serves to enhance membrane porosity and to create larger pore structures due to its preferential solvation effect, which promotes the formation of interconnected pore networks. Conversely, LiCl contributes to the overall thermal stability and mechanical strength of the membrane while facilitating the dissolution of PVDF, thereby improving the homogeneity of the polymer solution^31–34^.

Iron-nickel starfish-like (Fe_10_Ni_90_) filler have been demonstrated to form in porous/membrane-relevant contexts and to exhibit magnetic and thermal behaviors conducive to photothermal/desalination applications^26,35,36^. This work is contextualized within the broader framework of photothermal magnetic Janus membranes for solar-driven floating desalination, where magnetic fillers can influence buoyancy, heat localization, and salt handling^36^. Also, other researches provide a mechanistic basis for anticipating enhanced or modulated evaporation in pore-confined systems when magnetically active alloys are introduced^37,38^. In this study, however the filler is hydrophilic but included inside the hydrophobic polymeric matrix by small amount. The filler coated by the polymer and do that there is no direct contact between the water vapor and the filler but the filler help in local heating the attracted water vapor that affect positively in water vapor transportation from the feed side to the permeate side.

The Fe_10_Ni_90_ alloy was produced via a chemical reduction method as described in our previous work^26^, yielding distinct morphologies (starfish-like) suitable for dispersion in polymer matrices. The synthesized Fe_10_Ni_90_ alloy exhibits exceptional purity (approximately 99.9%) and robust stability, owing to the very low content of rapidly oxidizing iron in the mixture, which imparts strong magnetic characteristics and a high coercivity (Hc ≈ 106 emu/g). The particle size around 379 ± 50 nm and 246 ± 50 nm particle size with and without cones/needles was obtained. This combination enables its use in several applications. The selection of the iron–nickel alloy concentration was based on our previous work^7^ involving graphene nanoplates, which confirmed that the filler concentration should not exceed 0.2% to effectively assess its impact on membrane performance.

### Membrane thickness and filler effects

In MD, membrane thickness is a critical parameter that dictates the balance between mass transport efficiency and mechanical integrity. Generally, an increase in membrane thickness leads to higher mass transfer resistance, which directly hinders water vapor transport and consequently reduces permeate flux^39^. Furthermore, thicker membranes typically exhibit lower thermal conductivity, which weakens the crucial temperature gradient across the membrane necessary for efficient phase change and distillation. The specific membrane compositions listed in Table 1 detail the codes and components of the fabricated membranes.

**Fig. 2:**
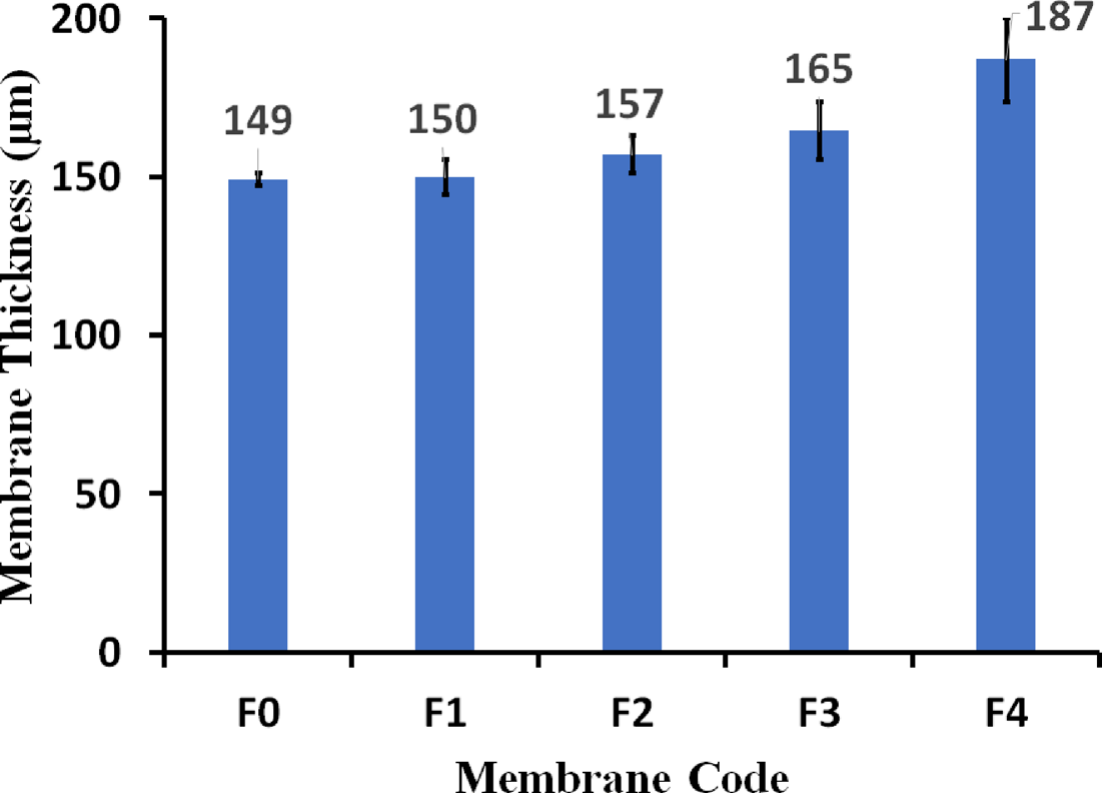
Membrane thickness (µm) as a function of membrane code. The standard deviation was calculated and added as error bar.

The thickness of the membranes was measured from three different samples prepared from distinct dope formulations, and standard deviation bars were included in all measurements to accurately represent variability. The interplay between membrane thickness and composition is essential for performance optimization. The incorporation of fillers, such as our magnetic iron–nickel alloy, can influence the final membrane thickness. For instance, membranes F1, F2, and F3 contain varying amounts of the alloy, which can contribute to a slight increase in thickness. Notably, membrane F3 (0.2 wt% alloy) showed only a modest 10% increase in thickness compared to the pristine (F0) as shown in Fig. 2, suggesting that at low filler loadings, the effect on thickness is minimal^40^. Conversely, changes in the polymer matrix, such as in membrane F4 (18 wt% polymer), result in a more significant thickness increase, up to 25% in this case. This increased thickness often correlates with enhanced mechanical stability, a desirable trait for long-term operation and resistance to physical stresses^41^.

The presence of additives, like LiCl, in the PVDF matrix can modulate ionic behavior and potentially improve thermal conductivity, which may partially offset the negative mass transport impact of greater thickness^3^. Achieving an optimal design, therefore, requires a careful trade-off: reinforcing structural integrity through filler loading must be weighed against the inevitable reduction in mass transport efficiency and potential increases in energy requirements associated with thicker membranes. The variations observed across membranes F0–F4 underscore the importance of a nuanced design strategy in MD applications, where maximizing flux should be harmonized with maintaining robust mechanical and structural properties^41^.

### Surface roughness

**Fig. 3:**
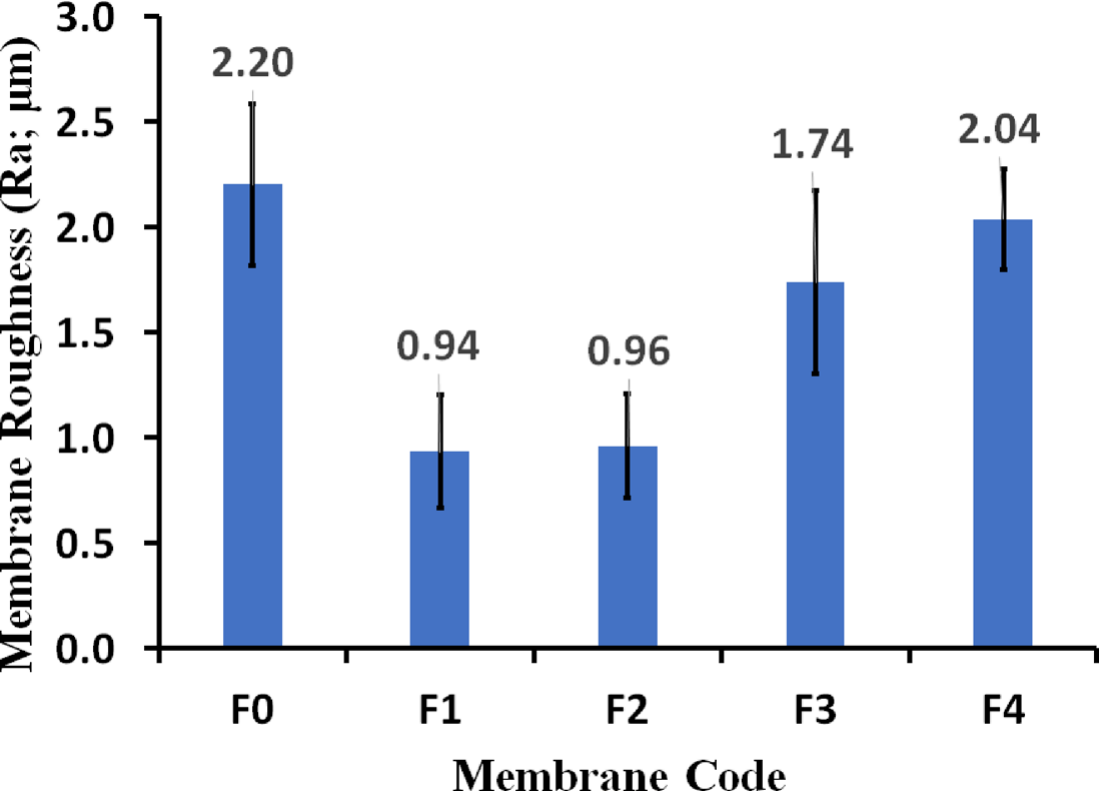
Membrane roughness (µm) as a function of membrane code. The standard deviation was calculated and added as error bar.

Surface morphology, particularly roughness, is a critical factor influencing the performance of hydrophobic membranes in MD. Generally, a smoother, more hydrophobic surface is less prone to liquid retention, which directly reduces the risk of pore wetting—the primary cause of flux decline and performance instability in MD^42^. Our initial observation—that incorporating the iron–nickel alloy reduced surface roughness as shown in Fig. 3 from 2.20 ± 0.39 μm to 0.94 ± 0.27 μm. Then, an increase in roughness to 1.74 ± 0.43 μm when raising the filler content from 0.1 wt% to 0.2 wt% suggests that at higher filler loadings, the alloy particles may interfere with the polymer precipitation in a way that promotes a less uniform, rougher surface morphology^43,44^. The observation that increasing polymer concentration can increase membrane roughness (2.04 ± 0.43 μm) is often linked to the kinetics of the Non-Solvent Induced Phase Separation (NIPS) process used in membrane fabrication. Higher polymer concentration leads to a more viscous dope solution, which slows the solvent/non-solvent exchange rate. This can result in less controlled precipitation, leading to the formation of larger, more irregular surface features, thus increasing the measured surface roughness^44^. The key remains optimizing the filler concentration to achieve the desired structural integrity (like mechanical stability) while maintaining a surface morphology that maximizes hydrophobicity and minimizes vapor transport resistance. However, the relationship between roughness and performance is complex and depends on the specific membrane structure. While a very smooth surface minimizes resistance, excessive smoothness or, conversely, extremely high roughness can lead to trade-offs. For instance, extremely high roughness can sometimes be associated with lower Liquid Entry Pressure (LEP) if the surface architecture is not hierarchically or re-entrant structured to trap air pockets effectively^43,44^.

Surface roughness, as described by Wenzel’s model, enhances the intrinsic wettability, meaning it enhances both hydrophilicity and hydrophobicity^45^. At higher filler loadings (F3), the morphology of the iron-nickel alloy particles might lead to increased surface roughness or a change in the surface texture that favors a more hydrophobic state (Cassie-Baxter state) over the initial Wenzel state, or simply that the roughness effect begins to dominate over the chemical effect of the additive itself^45,46^. Clearly, at higher concentrations (F3), the additive particles may start to agglomerate or orient themselves in a way that shields the most hydrophilic features exposed at low concentrations, or they might create a rougher surface structure that traps air pockets, leading to a higher apparent contact angle^45^. For that, the non-monotonic behavior is likely a result of a transition where the initial chemical effect of the low-concentration additive (increasing hydrophilicity) is gradually superseded by morphological effects like increased surface roughness or changes in particle dispersion/orientation at higher concentrations, which can enhance the overall apparent hydrophobicity^45,46^.

### Membrane porosity

In MD systems, membrane porosity is a key factor governing performance, directly influencing both permeability and separation efficiency. While high porosity enhances permeation rates, it can also increase the risk of wettability, potentially compromising overall membrane efficiency. The fabricated nanocomposite membranes incorporating magnetic nanoparticles exhibit significant improvements in porosity. For example, the pristine PVDF membrane (F0) displays a porosity of 54.38 ± 2.7%, whereas the addition of 2%wt. alloy/filler in membrane F3 increases porosity markedly to 74.32 ± 3.7% (Table 2). Interestingly, further increasing the polymer content in F4 does not lead to substantial changes in porosity compared to membranes with similar filler content, as seen in the comparable values for F1 and F4 (63.78 ± 3.2). This underscores the nuanced interplay between filler concentration, polymer content, and membrane structural integrity.

The data show in Table 2a general inverse relationship between porosity and LEP among the membranes, where higher porosity tends to correspond to lower LEP, suggesting that more open structures offer easier pathways for liquid penetration. For example, as porosity rises from 54.38 ± 2.7% (F0) to 74.32 ± 3.7% (F3), LEP declines from about 80 ± 4 kPa to 70 ± 2 kPa, indicating reduced resistance to water entry. However, the F4 sample disrupts this simple trend: with a porosity of 63.78 ± 3.2% but a markedly higher LEP of 84 ± 4 kPa, it implies that factors beyond overall porosity—such as surface wettability, or the specific filler/polymer interactions—strongly influence LEP. Thus, while porosity is a useful indicator, LEP is governed by a combination of structural and interfacial properties.

**Table 2:** Membrane porosity (%) and Liquid Entry Pressure (LEP) of the fabricated membranes. The standard deviation was calculated and added.

Membrane Code	Alloy/filler content	Porosity (%)	LEP (kPa)
**F0**	0	54.38 ± 2.7	80 ± 4
**F1**	0.01	65.05 ± 3.2	75 ± 2
**F2**	0.1	66.52 ± 3.3	73 ± 3
**F3**	0.2	74.32 ± 3.7	70 ± 2
**F4 (18 wt% polymer)**	0.01	63.78 ± 3.2	84 ± 4

### Membrane wettability

**Fig. 4:**
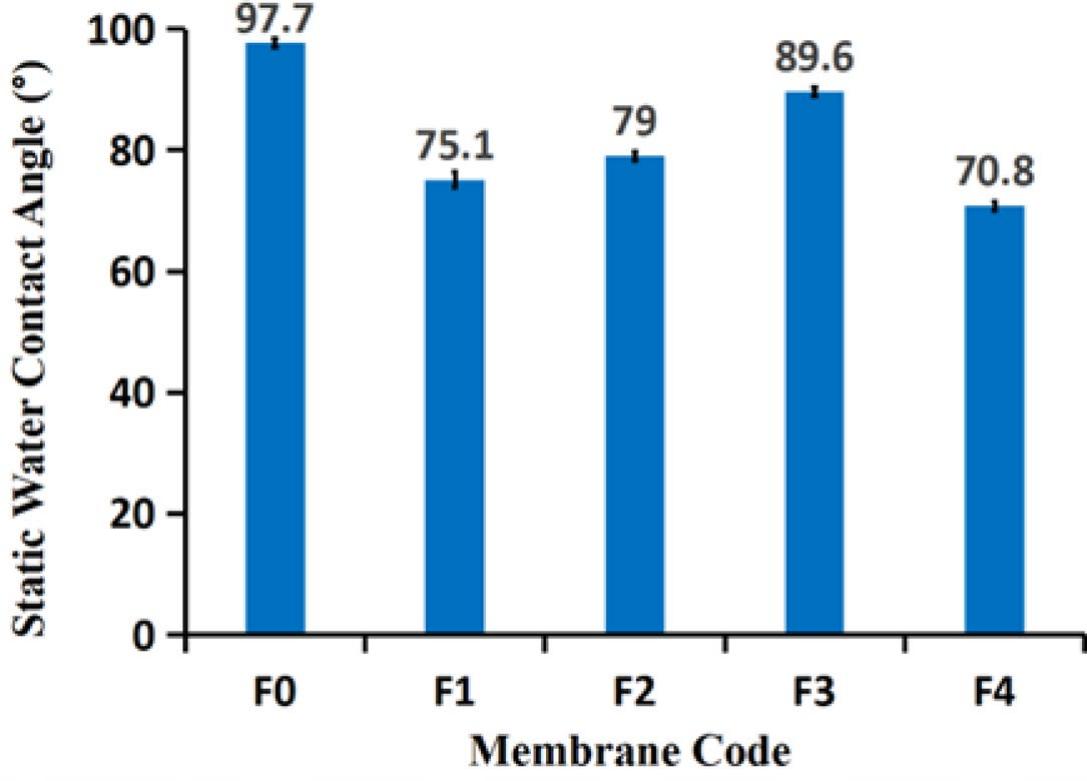
The static water contact angle of different fabricated membranes. The standard deviation was calculated and added as error bar.

The interplay between membrane thickness, roughness, and porosity is critical for optimizing performance in membrane distillation. While higher porosity generally enhances permeability, it can also increase surface roughness, influencing the static water contact angle, as illustrated in Fig. 4. The pristine membrane (F0) exhibits a high contact angle of 97.7 ± 0.7°, reflecting its hydrophobic nature. Incorporation of a low filler concentration (F1) reduces the angle to 75.1 ± 1.3°, indicating increased hydrophilicity. With moderate filler content (F2), the contact angle rises slightly to 79.0 ± 0.7°, suggesting a partial return to hydrophobic behavior. At higher filler loading (F3), the angle further increases to 89.6 ± 0.6°, likely due to enhanced surface roughness or stronger hydrophobic interactions, highlighting the complex relationship between surface texture and wettability. In contrast, F4, combining lower filler content with higher polymer concentration, shows a contact angle of 70.8 ± 0.7°, demonstrating how polymer composition can strongly influence membrane hydrophobicity.

This intricate interplay between porosity, thickness, and roughness underscores the importance of careful design in developing high-performance nanocomposite membranes for membrane distillation^47^. Achieving optimal membrane performance requires a delicate balance, ensuring that increased porosity enhances permeability without compromising structural integrity or functional efficiency, as high porosity is generally linked to higher flux but also potential thermal losses and mechanical vulnerability^48^. Understanding and controlling these parameters is therefore essential for guiding future advancements in membrane technology for distillation applications^49^, where thinner membranes can increase flux but decrease mechanical strength, and filler incorporation should be optimized to maintain a favorable surface morphology for anti-wetting properties^47–49^.

### Mechanical properties

The filler/iron–nickel alloy content has a significant impact on membrane mechanical performance, which is critical for membrane distillation. Increasing filler content enhances tensile strength, as evidenced by the rise from 7.664 MPa in F0 to 17.716 MPa in F3 (Table 3), reflecting improved structural integrity. Strain behavior, however, is more complex: it decreases at low filler content (F1) but increases at higher loadings (F3), indicating that while the filler reinforces strength, it also affects flexibility. Achieving an optimal balance is essential, as higher strength improves durability and thermal stability—key factors for MD operation—while sufficient strain ensures the membrane can accommodate thermal expansion and operational fluctuations^50^. Careful optimization of filler content is therefore crucial to maximize both mechanical robustness and functional performance^51^.

**Table 3:** The mechanical properties (Strength and Strain) of the fabricated membranes. The standard deviation was calculated and added.

Membrane Code	Stress (MPa)	Strain (%)
F0	7.7 ± 0.4	59 ± 3
F1	13.3 ± 0.6	44 ± 2
F2	14.4 ± 0.7	58 ± 3
F3	17.8 ± 0.9	62 ± 3
F4 (18%polymer)	13.3 ± 0.7	44 ± 2

### Morphology observed by SEM

**Fig. 5:**
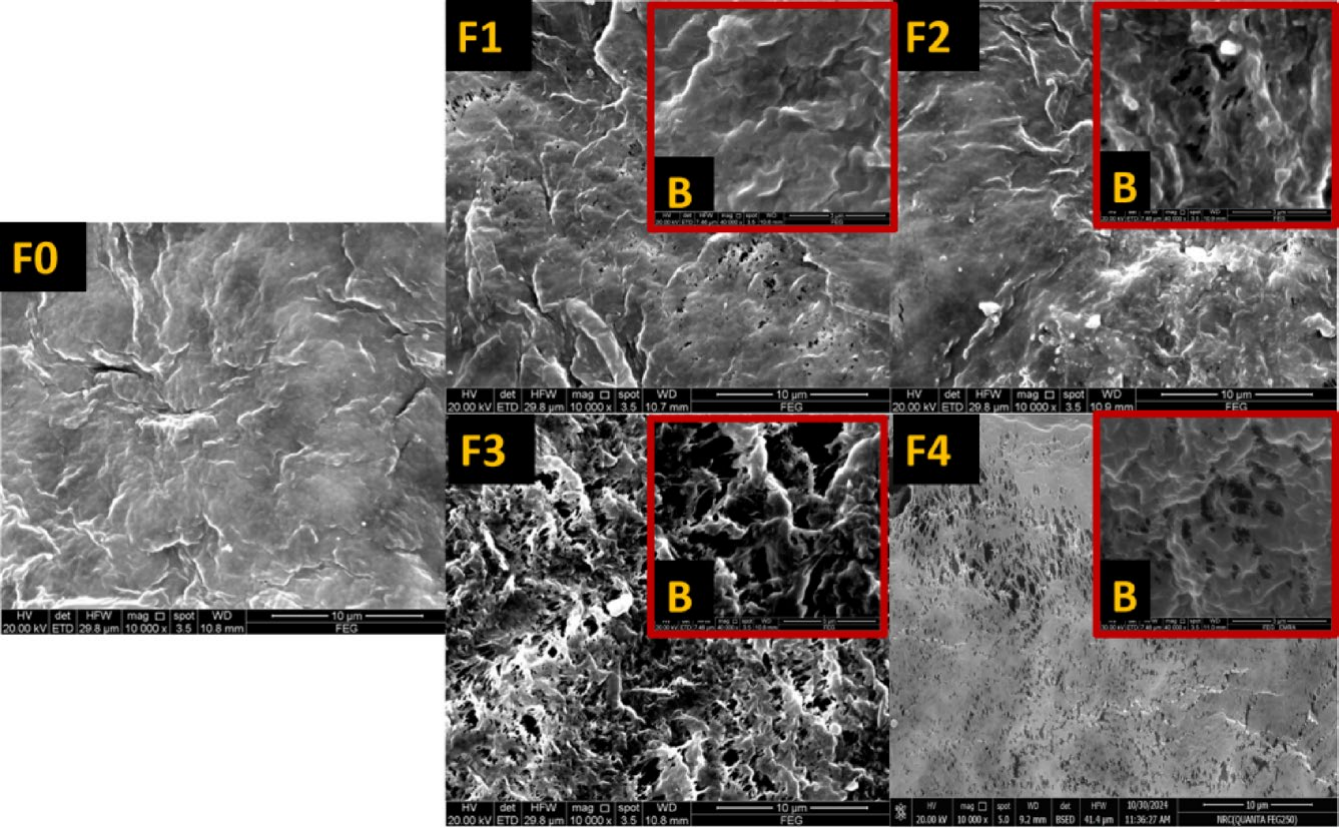
SEM images show the surface membrane morphology of the fabricated membranes at magnifications of ×10,000 and ×40,000 (B).

Figure 5 presents a comparative analysis of membrane morphologies (F0–F4) using scanning electron microscopy (SEM) at magnifications of ×10,000 and ×40,000, highlighting the effects of varying polymer content and iron–nickel alloy/filler on surface characteristics and porosity. The control membrane F0 (pristine PVDF) exhibits a relatively smooth surface with limited texture and a porosity of 54.38%, providing fewer pathways for vapor transport and potentially constraining productivity in MD applications.

Introducing a small amount of alloy/filler in F1 (0.01 wt% and 65.05% porosity) begins to modify the membrane morphology, creating subtle textural irregularities that slightly enhance pathways for vapor transport, resulting in modest improvements in permeate flux. F2, containing 0.1 wt% alloy/filler, shows a more pronounced increase in surface roughness and slightly higher porosity (66.52%), facilitating improved vapor movement and membrane efficiency^52^.

Membrane F3 demonstrates the most significant morphological transformation, with pronounced surface roughness and texture, achieving a porosity of 74.32%. These features markedly increase vapor transport p athways, supporting enhanced permeate flux and confirming that higher porosity can improve MD productivity. In contrast, F4, with higher polymer content (18 wt%) and a porosity of 63.78%, reflects a balance between surface roughness and increased thickness. While the intermediate porosity maintains reasonable vapor transport, the added thickness contributes to mechanical stability, highlighting the trade-off between structural integrity and flux^53^.

Overall, the SEM images underscore the complex interplay between porosity, roughness, and thickness in determining membrane performance. They clearly illustrate how variations in polymer composition and alloy/filler content can fine-tune these structural parameters, optimizing membranes for enhanced efficiency in membrane distillation applications^54^.

The pore size distribution of the pristine PVDF (F0) and F3 membranes was estimated from SEM micrographs using ImageJ program based on the scale bar calibration. More than 1000 pores were measured to obtain reliable statistical data. Outliers were removed using the interquartile range method prior to analysis. The image of F0 reveals a notably dense and compact surface morphology characterized by a sparse distribution of pore openings across the membrane surface. The majority of the observed pores are small and relatively uniform, indicating a predominance of a narrow pore size distribution. According to the SEM analysis, the pore diameters of the F0 membrane are estimated to range from approximately 0.05 to 0.25 μm, with an average size of about 0.14 ± 0.01 μm. This relatively diminutive pore size and tight distribution imply limited pore development during the phase inversion process. The results of iron-nickel mixed PVDF matrix (F3) revealed that the pore diameters ranged from 0.106 μm to approximately 3.66 μm, with an average pore size of 0.34 ± 0.03 μm. The majority of pores (≈ 70%) were distributed within the 0.1–0.4 μm range, indicating a highly microporous structure. This porous morphology facilitates vapor transport while maintaining liquid entry pressure, which is beneficial for membrane distillation applications.

### Energy dispersive X-ray spectroscopy (EDX) analysis

**Fig. 6:**
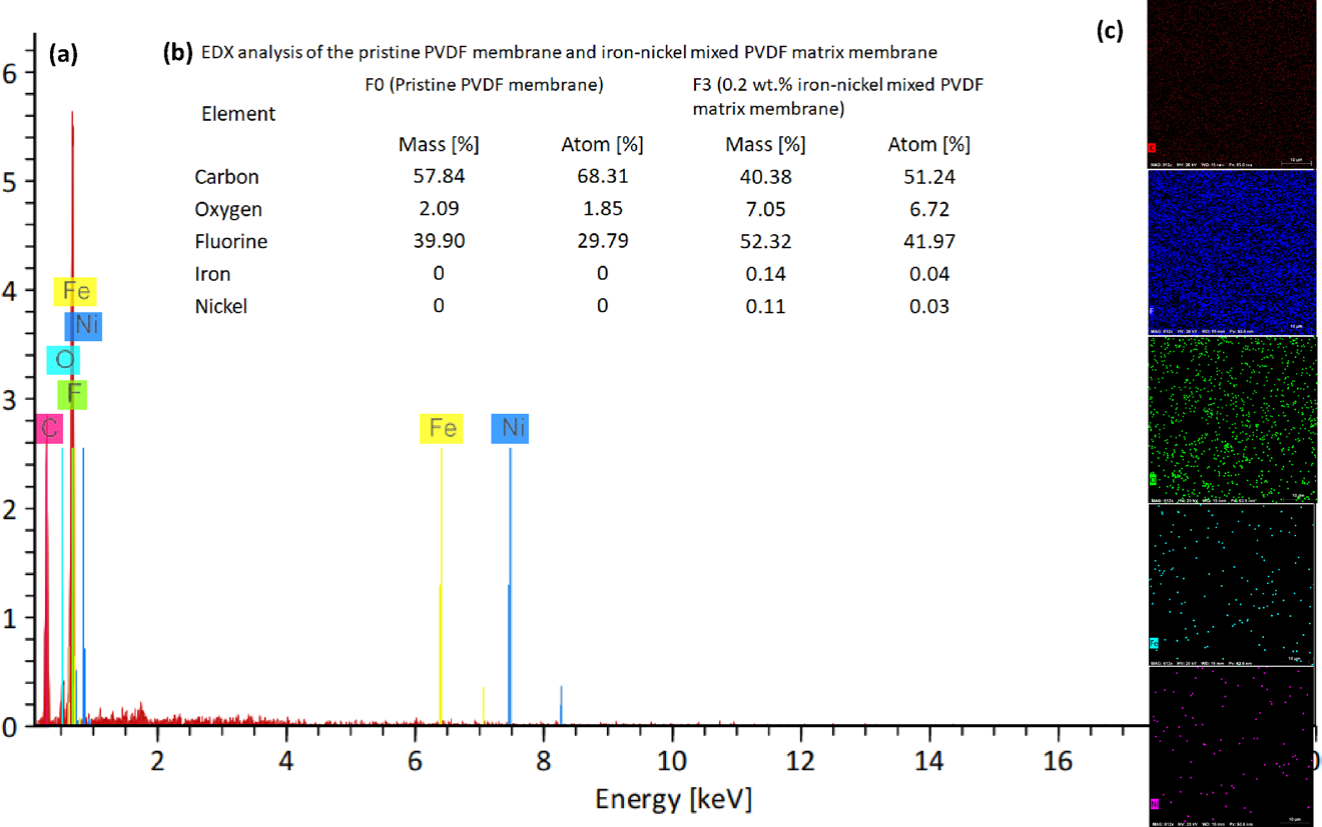
Energy Dispersive X-ray Spectroscopy (EDX) analysis of both the pristine PVDF membrane; F0, and iron-nickel mixed PVDF matrix membrane; F3. **(a)** elemental composition chart and **(c)** elemental mapping of F3. **(b)** The elemental analysis of both F0 and F3.

Figure 6 presents a detailed Energy Dispersive X-ray Spectroscopy (EDX) analysis of both pristine PVDF membranes and iron-nickel mixed PVDF matrix membranes. Panel (a) showcases the elemental composition, highlighting the presence of Carbon (C), Oxygen (O), Fluorine (F), Iron (Fe), and Nickel (Ni) with corresponding mass and atomic percentages. Notably, the pristine PVDF membrane displays a significant predominance of Carbon and Fluorine, reflecting its chemical makeup. In contrast, the iron-nickel mixed membrane exhibits iron and Nickel percentages, which confirm the metal incorporation. Furthermore, panel (c) features elemental mapping, illustrating the spatial distribution of these elements across the membrane; this visual representation is crucial in assessing the uniformity of the metal mixing and its potential effects on membrane performance. Overall, the figure emphasizes the compositional differences and their implications for the functionality of PVDF-based membranes.

### Fourier Transform Infrared equipped by Attenuated Total Reflection (FTIR-ATR)

**Fig. 7:**
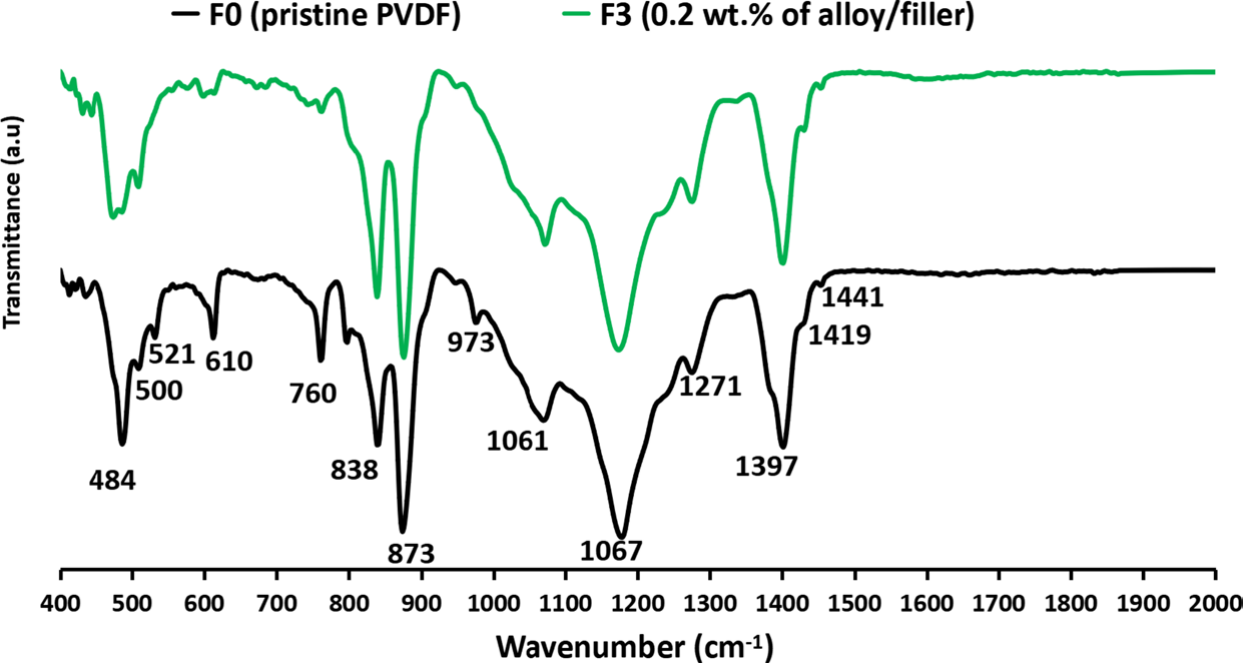
Fourier Transfer Infrared spectroscopy-Attenuated Total Reflectance (FTIR-ATR) analyses of the pristine poly(vinylidene fluoride) (PVDF) membrane and the magnetic mixed PVDF matrix; F3, 0.2 wt% of iron-nickel alloy/filler.

As shown in Fig. 7, the FTIR spectra of the PVDF membranes exhibit characteristic bands that reflect the polymer’s crystalline phases. Peaks at 760, 873, and 1067 cm⁻¹ correspond to the α-phase, associated with specific molecular vibrations within the α-PVDF crystalline structure. Bands at 838, 1271, 1419, and 1441 cm⁻¹ are indicative of the β-phase^35^. Notably, the reduction in intensity of the 610 cm⁻¹ and 760 cm⁻¹ peaks—linked to C–C and C–H bending vibrations, respectively—upon addition of the iron–nickel alloy/filler suggests significant structural modifications. In membrane F3, this diminishment may indicate a partial phase transition from α-PVDF to β-PVDF, as the alloy alters the crystalline arrangement and increases amorphous regions^55,56^. Furthermore, the formation of new intermolecular interactions, such as hydrogen bonding^57,58^, could influence these vibrational modes, highlighting the role of the iron–nickel alloy/filler in modulating the molecular structure and properties of the PVDF membranes.

### Thermal ravimetric analysis (TGA)

**Fig. 8:**
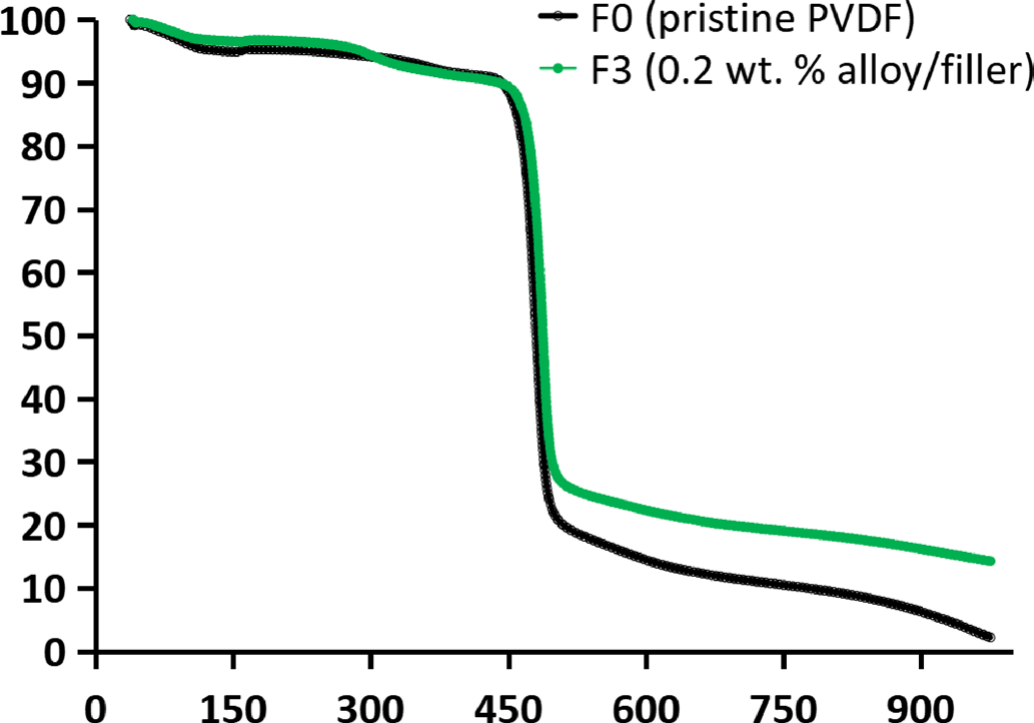
Thermal gravimetric analysis (TGA) of the pristine poly(vinylidene fluoride) (PVDF) membrane (F0) and the magnetic mixed PVDF matrix; F3, 0.2 wt% of iron-nickel alloy/filler.

Thermogravimetric (TGA) and derivative thermogravimetric (DTG) analyses were conducted to assess the effect of Fe–Ni alloy/filler incorporation on the thermal stability of PVDF membranes. As shown in Fig. 8, the pristine PVDF membrane (F0) exhibited an initial minor mass loss of ~ 0.95% between 30 and 40 °C, attributed to the release of adsorbed moisture and residual solvent. The primary decomposition occurred between ~ 430–520 °C, with a DTG peak at 468.7 °C, corresponding to thermal scission of the PVDF backbone. The residual mass at 1000 °C was low (~ 5–6%), reflecting the predominantly organic composition of the membrane^35,59^.

In contrast, the Fe–Ni–loaded membrane (F3) demonstrated enhanced thermal resistance. The main degradation step shifted to higher temperatures, with a DTG peak at ~ 486–490 °C (not showed here), indicating delayed decomposition. The broader degradation peak suggests more gradual thermal breakdown. Notably, the final residue increased to ~ 13–15% at 1000 °C, significantly higher than F0, highlighting the contribution of thermally stable Fe–Ni alloy/filler. These Fe–Ni alloy/filler likely act as heat-absorbing centers and diffusion barriers, restricting polymer chain mobility and slowing the escape of volatile degradation products. The higher residue also suggests enhanced char formation and the presence of inorganic components.

Overall, the incorporation of Fe–Ni alloy/filler substantially improves the thermal durability of PVDF membranes, rendering the PVDF/Fe–Ni mixed-matrix membrane more suitable for MD applications involving elevated temperatures, thermal regeneration, or prolonged exposure to hot vapor streams^59^.

### VMD performance of PVDF/Fe–Ni membranes

**Fig. 9:**
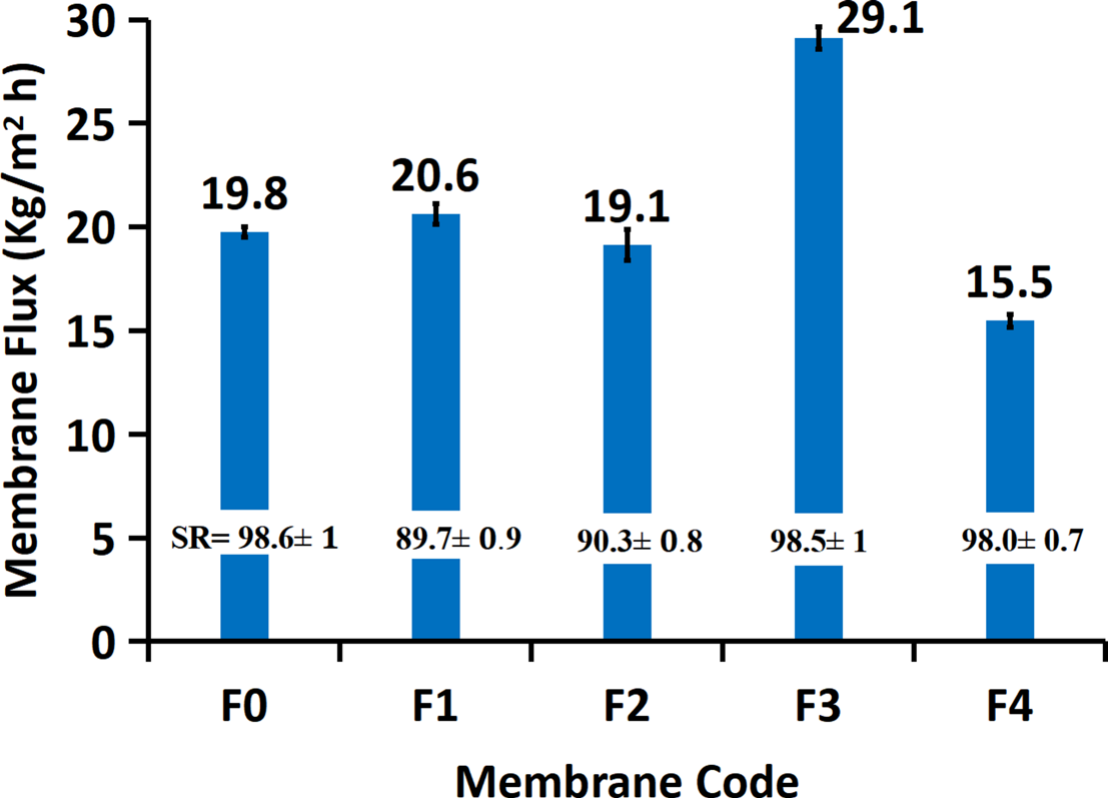
Membrane Flux and salt rejection (SR) of the prepared membranes using vacuum membrane distillation (VMD) setup. The standard deviation was calculated and added as error bar.

The vacuum membrane distillation (VMD) performance of the fabricated PVDF/Fe–Ni membranes is strongly influenced by their structural and surface properties. Figure 9 presents the flux and salt rejection (SR) for membranes F0–F4, highlighting the role of morphology, porosity, roughness, thickness, and hydrophilicity in water transport efficiency. Among the membranes, F3 exhibits the highest flux at 29.1 kg/m²·h, significantly surpassing the other formulations, which range from 15.5 kg/m²·h for F4 to 20.6 kg/m²·h for F1. This superior performance is primarily attributed to F3’s optimized combination of high porosity, favorable thickness, and controlled surface roughness, which collectively enhance permeability while maintaining selectivity. High porosity facilitates greater vapor transport pathways, directly enhancing flux. Additionally, the incorporation of nanoparticles like magnetite^59^ as example in a PVDF matrix can enhance flux by over 36% in Direct Contact Membrane Distillation (DCMD), often by rendering the membrane bulk more hydrophilic while maintaining a hydrophobic top surface, thus increasing water uptake. In contrast, F4 demonstrates the lowest flux, suggesting that its higher polymer content and smoother surface may limit water transport despite improved mechanical stability.

Surface roughness and wettability are crucial in mediating water vapor flux in VMD. Membrane F3’s high flux suggests a more favorable balance in its contact angle profile, facilitating water vapor passage and reducing resistance to flow. Conversely, the smoother surface of F4 may impede water vapor transport, contributing to its lower flux. The effect of roughness is complex. While molecular dynamics simulations indicate that roughness can increase drag resistance on hydrophilic surfaces, for hydrophobic surfaces, roughness may enhance apparent slip velocity and flow. The observation that F3’s increased roughness improves water vapor transport suggests it is conducive to enhanced water vapor creation and transport, optimizing the vapor-liquid interface within the pores^60,61^.

The observed ~ 10% increase in membrane thickness with a 0.2 wt% filler loading is consistent with expectations, indicating that the filler was well-dispersed throughout the matrix without any significant accumulation. This minimal change in thickness reflects the effective integration of the filler into the membrane structure, which is vital for maintaining the desired properties while enhancing performance. Our findings confirm the uniform distribution of the filler, thereby supporting the reproducibility and reliability of the results presented in this study. Thinner membranes generally reduce the mass transfer resistance, leading to higher flux. As membrane F3 is thinner than F4 (Fig. 2), this factor would contribute to its superior performance, assuming other factors are comparable. However, thickness must be balanced against the need for structural integrity. Notably, studies comparing PVDF membranes in membrane crystallization suggest that a thicker membrane may increase resistance to mass transport while decreasing heat loss^62^.

The inclusion of magnetic $\:\mathrm{Fe-Ni}\text{}$alloy/filler introduces a potential mechanism for flux enhancement. The magnetic field or the presence of the magnetic material itself may influence the evaporation rate within the pores by potentially disrupting hydrogen bonds in water clusters at the water-air interface. This is supported by literature^63–65^ indicating that increasing the concentration of magnetic nanoparticles in various membrane processes can significantly enhance water vapor flux.

Hydrophilicity, as indicated by water contact angle measurements, further correlates with flux behavior. A lower contact angle promotes water uptake and transport, while higher angles reduce wettability. Membrane F3 likely exhibits a more favorable contact angle, aligning with its superior flux, whereas F4’s characteristics, including a suboptimal contact angle and higher polymer content, may increase water interaction at the surface in a way that limits efficient transport^66^.

From an alternative perspective, the influence of magnetic iron-nickel alloy/filler may enhance the evaporation rate of water within the membrane’s porous structure. This magnetization potentially impacts the disruption of hydrogen bonds within water clusters especially at water-air interface, as demonstrated in previous studies^66–68^. The F3 membrane shows a significantly different surface morphology characterized by a highly porous and heterogeneous structure with numerous interconnected pores. The pore openings are larger and more abundant compared with those of the pristine membrane, leading to a broader pore size distribution. The incorporation of iron-nickel alloy/filler enhance the thermodynamic instability of the polymer solution during phase inversion, accelerating solvent–nonsolvent exchange and promoting the formation of larger pores and interconnected channels. Consequently, the F3 membrane demonstrates higher pore density and larger average pore size relative to F0.

Notably, membrane F3 shows around 70% of the pores fall within the 0.1–0.4 μm range, indicating a highly microporous structure that enhances vapor transport while effectively preventing liquid entry, exemplified by a liquid entry pressure (LEP) of 70 ± 2 kPa. With a porosity of 74.32 ± 3.7%, this configuration facilitates a measured flux of 29.1 kg/m².h, demonstrating the membrane’s capacity to optimize performance in membrane distillation applications. The interplay between small pore sizes, high porosity, and suitable LEP underscores the membrane’s potential for efficient separation processes. In terms of salt rejection, the varying flux rates point towards potential trade-offs between permeability and salt exclusion. The data clearly indicate that within the context of VMD, membrane F3 offers a promising combination of high flux and effective salt rejection. However, the 89.7 ± 0.9% rejection suggests that while the process is largely vapor-driven, there is a measurable degree of salt transport, likely due to partial wetting or the inherent characteristics of the membrane structure under the operating conditions. High salt rejection for membrane F3 indicates that the incorporation of “Fe-Ni” did not compromise the essential hydrophobic nature of the PVDF matrix at the vapor transport surface. Membrane F3 represents an optimal configuration where structural factors (porosity and thickness) and the influence of the $\:\mathrm{Fe-Ni}$ filler synergize to maximize VMD flux while maintaining effective salt rejection. Future work should focus on long-term stability testing, quantifying the precise role of the magnetic field effect on the water-air interface, and optimizing the $\:\mathrm{Fe-Ni}\text{}$loading to further enhance the roughly 47% flux increases without compromising the high salt rejection of about 98.5 ± 1.0%.

Overall, these results demonstrate the intricate interplay between roughness, thickness, porosity, and wettability in dictating membrane performance in VMD. Membrane F3 exemplifies a well-balanced configuration of these parameters, achieving optimal flux and salt rejection. In contrast, F4 illustrates how deviations in these properties, such as excessive polymer content or suboptimal surface characteristics, can hinder performance. Understanding and fine-tuning these interrelated factors is therefore critical for the rational design of high-efficiency membranes for desalination applications.

## Overall discussion

Increasing polymer content in PVDF membranes exerts a pronounced influence on porosity, which in turn directly affects productivity in MD. As shown in Table 2, the porosity of the fabricated membranes increases with the incorporation of Fe–Ni alloy/filler, rising from 54.38% in the pristine membrane F0 to 74.32% in F3, which contains 0.2 wt% alloy. Higher porosity provides more pathways for vapor transport, thereby enhancing permeate flux and overall membrane productivity. The interplay between surface roughness, membrane thickness, and polymer content further modulates porosity and performance. For example, F4, with 18 wt% polymer and only 0.01 wt% alloy, exhibits a porosity of 63.78%, lower than that of F2 and F3. Nevertheless, the balance between roughness and thickness remains critical: while increased thickness improves mechanical stability, it can also introduce additional resistance to vapor flow if not carefully optimized alongside porosity. Similarly, surface roughness influences wettability and flow resistance, underscoring the need for a finely tuned combination of structural parameters. Consequently, the synergy among porosity, roughness, and thickness fundamentally determines the membrane’s ability to facilitate efficient vapor transport in VMD. While higher porosity generally enhances productivity, excessive roughness or thickness can counteract these gains. Optimizing polymer content is therefore essential to achieve an equilibrium that maximizes both porosity and overall membrane performance.

In VMD, the permeate is expected to contain a negligible amount of dissolved salts because the transfer mechanism is primarily vapor transport through the membrane pores, while liquid-phase transport should be prevented by the hydrophobic nature of the membrane. However, a small salt presence in the permeate can still occur in practice. This is commonly attributed to factors such as slight membrane wetting (partial penetration of the feed liquid into the membrane pores), concentration polarization at the membrane–feed interface, and possible carryover of entrained feed droplets or salt-containing aerosols under unstable vapor–liquid dynamics. In addition, the experimental conditions (e.g., transmembrane pressure, feed salinity, and operating temperature) may promote local changes in pore wetting behavior, leading to residual salt in the permeate. Therefore, the observed permeate salinity is not indicative of predominant liquid transport, but rather reflects minor mass-transfer losses associated with the above mechanisms.

A particularly noteworthy outcome of this study is the synergistic effect of the membrane structure combined with the magnetic properties of the Fe–Ni alloy/filler. The magnetic component not only reinforces structural integrity but also promotes water evaporation within the membrane pores, contributing to increased flux without significantly compromising salt rejection. This highlights the potential of integrating functional fillers to simultaneously optimize both structural and functional performance in MD membranes.

## Conclusions

This study demonstrates the critical importance of optimizing membrane properties to enhance performance in vacuum membrane distillation (VMD). The interplay among membrane thickness, roughness, and porosity plays a decisive role in governing permeate flux and salt rejection. Membrane F3 exemplifies an optimal balance of these parameters, achieving superior structural integrity and high permeability due to its elevated porosity (74.32%) and well-controlled surface roughness.

In contrast, formulations such as F4 highlight the potential drawbacks of excessive polymer content. While higher polymer loading can improve mechanical stability, it may reduce porosity, increase mass transfer resistance, and hinder flux, illustrating the need for a nuanced approach that balances mechanical strength, thermal efficiency, and transport properties.

The incorporation of magnetic Fe–Ni alloy further enhances membrane performance. Membranes containing 0.2 wt% alloy and 14% PVDF achieved the highest flux (29.1 kg/m²·h), representing a 47% improvement over the pristine PVDF membrane, without compromising salt rejection. This enhancement is attributed to the synergistic effects of the alloy, which reinforce structural integrity and promote water evaporation within the membrane pores. The critical interdependence of thickness, roughness, porosity, mechanical strength, wettability, and thermal stability in determining MD membrane performance. Careful tuning of polymer content and Fe–Ni filler incorporation enables the design of nanocomposite membranes that maximize flux and maintain structural integrity providing a robust framework for advanced MD membrane development.

In this work, we focus on establishing the principle and proof-of-concept of our proposed approach for desalination membranes. While we recognize the importance of long-term performance, fouling resistance and stability testing, these aspects are inherently time-intensive and are more suitably addressed in dedicated follow-up studies. The present manuscript provides the experimental framework, material/system characterization, and short-term performance data that validate the underlying mechanism and feasibility. We have outlined a clear plan for future work to extend the evaluation to longer-term aging (months), fouling scenarios with representative feed streams, wetting and stability under operational conditions, and repeated cleaning cycles. This staged approach allows us to rigorously establish reliability and scalability across subsequent publications, while delivering a solid principled contribution in the current work.

Overall, these findings underscore the necessity of careful membrane design, where porosity, roughness, and thickness are optimized in concert with functional additives such as magnetic alloys. Such a strategy enables the development of high-efficiency membranes for VMD, paving the way for more sustainable and effective desalination technologies.

## Data Availability

All data generated or analyzed during this study are included in this published article.
